# Epstein-Barr Virus-Positive Diffuse Large B-cell Lymphoma Arising in Ulcerative Colitis: An Instructive Case Report and Literature Review

**DOI:** 10.7759/cureus.72506

**Published:** 2024-10-27

**Authors:** Yuichiro Hamamoto, Saori Matsui, Kiyotaka Okawa, Chu Matsuda, Shin-ichi Nakatsuka, Hironao Yasuoka, Takayoshi Goto, Tsunekazu Mizushima

**Affiliations:** 1 Diagnostic Pathology, Osaka International Medical &amp; Science Center, Osaka, JPN; 2 Gastroenterology, Yodogawa Christian Hospital, Osaka, JPN; 3 Gastroenterological Surgery, Osaka International Medical &amp; Science Center, Osaka, JPN; 4 Pathology, Yao Tokushukai General Hospital, Yao, JPN; 5 Diagnostic Pathology, Osaka Rosai Hospital, Sakai, JPN; 6 Surgery, Dokkyo Medical University, Mibu, JPN

**Keywords:** epstein-barr virus, inflammatory bowel disease, lymphoma, tacrolimus, ulcerative colitis

## Abstract

Malignant lymphoma is a relatively rare complication in patients with ulcerative colitis (UC) and its etiology is unclear. We present a hard-to-diagnose case of Epstein-Barr virus-positive diffuse large B-cell lymphoma in an elderly patient with UC who was treated with the immunomodulator tacrolimus and herbal medicine including indigo naturalis. Because malignant lymphomas can mimic other inflammatory diseases macroscopically, diagnosis in such cases can be challenging.

## Introduction

Ulcerative colitis (UC), a chronic inflammatory bowel disease (IBD), often impacts young adults. While long-term treatment for UC carries a cancer risk, the incidence of malignant lymphoma in UC patients appears similar to the general population [1-3]. However, malignant lymphoma can be diagnostically challenging due to its ability to mimic other inflammatory diseases macroscopically.

The mainstay of IBD treatment includes 5-aminosalicylic acid, prednisolone, immunomodulators, and molecular-targeted therapies. Thiopurines, a class of immunomodulators that include azathioprine and 6-mercaptopurine, are associated with a significantly increased risk of malignant lymphoma, particularly Epstein-Barr virus (EBV)-positive lymphoma [2,4-8]. While anti-tumor necrosis factor-α therapy, a type of molecular-targeted therapy, has not shown a clear risk of lymphoma in IBD patients, its risk assessment is complicated by its frequent co-administration with thiopurines [9-11].

We present a case of an elderly patient with UC who developed EBV-positive diffuse large B-cell lymphoma (EBV-positive DLBCL) after receiving treatment with immunomodulator tacrolimus and herbal medicine including indigo naturalis.

## Case presentation

Clinical course

**Figure 1:**
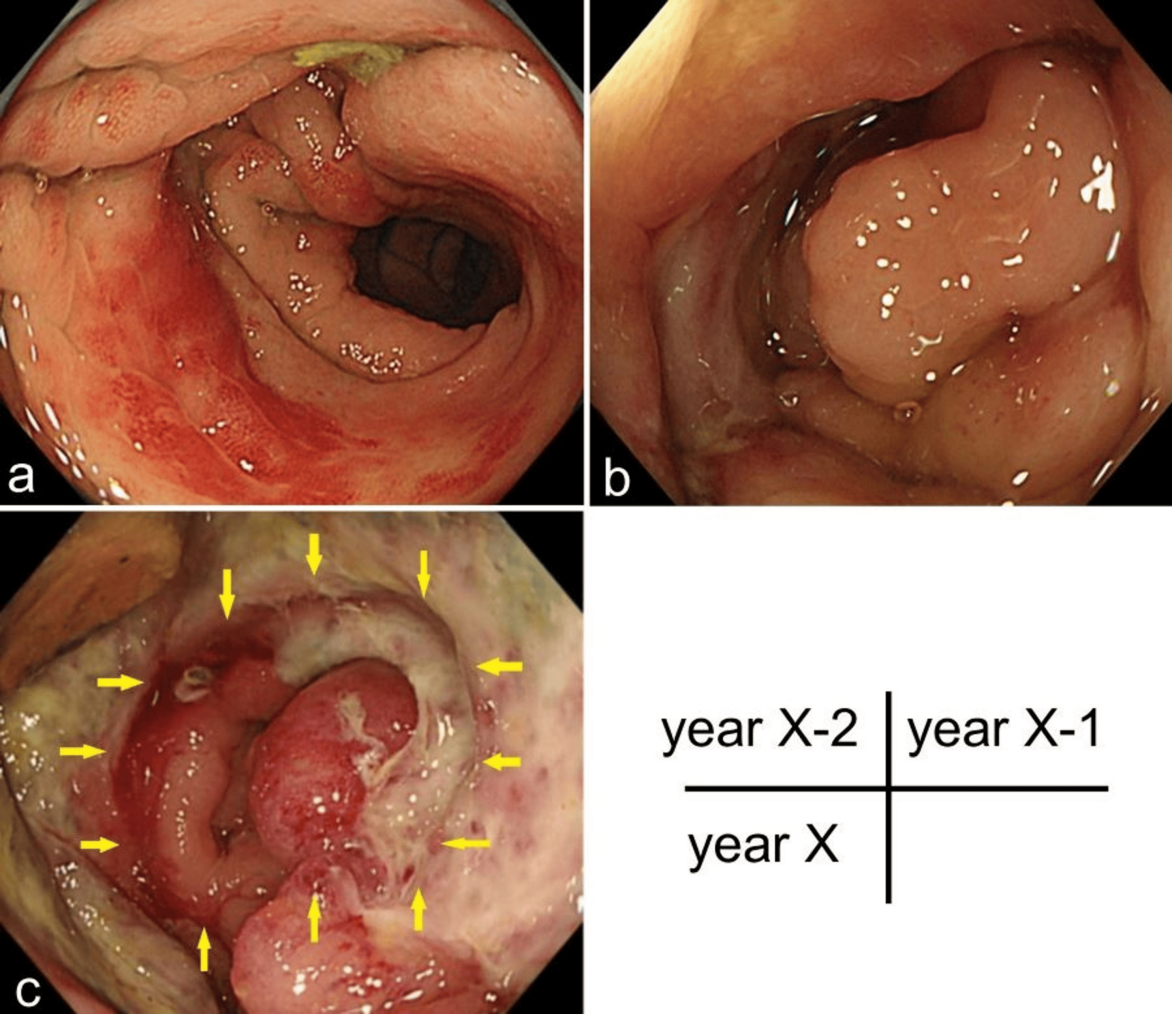
Endoscopic images of the sigmoid colon Mucosal edema and intramucosal bleeding were observed, accompanied by edematous stenosis. (a) No obvious erosions or ulcers were observed (year X-2). (b) Mucosal edema, stenosis, and deep ulcers were observed (year X-1). (c) Punched-out ulcer was seen (year X, yellow arrow).

**Figure 2:**
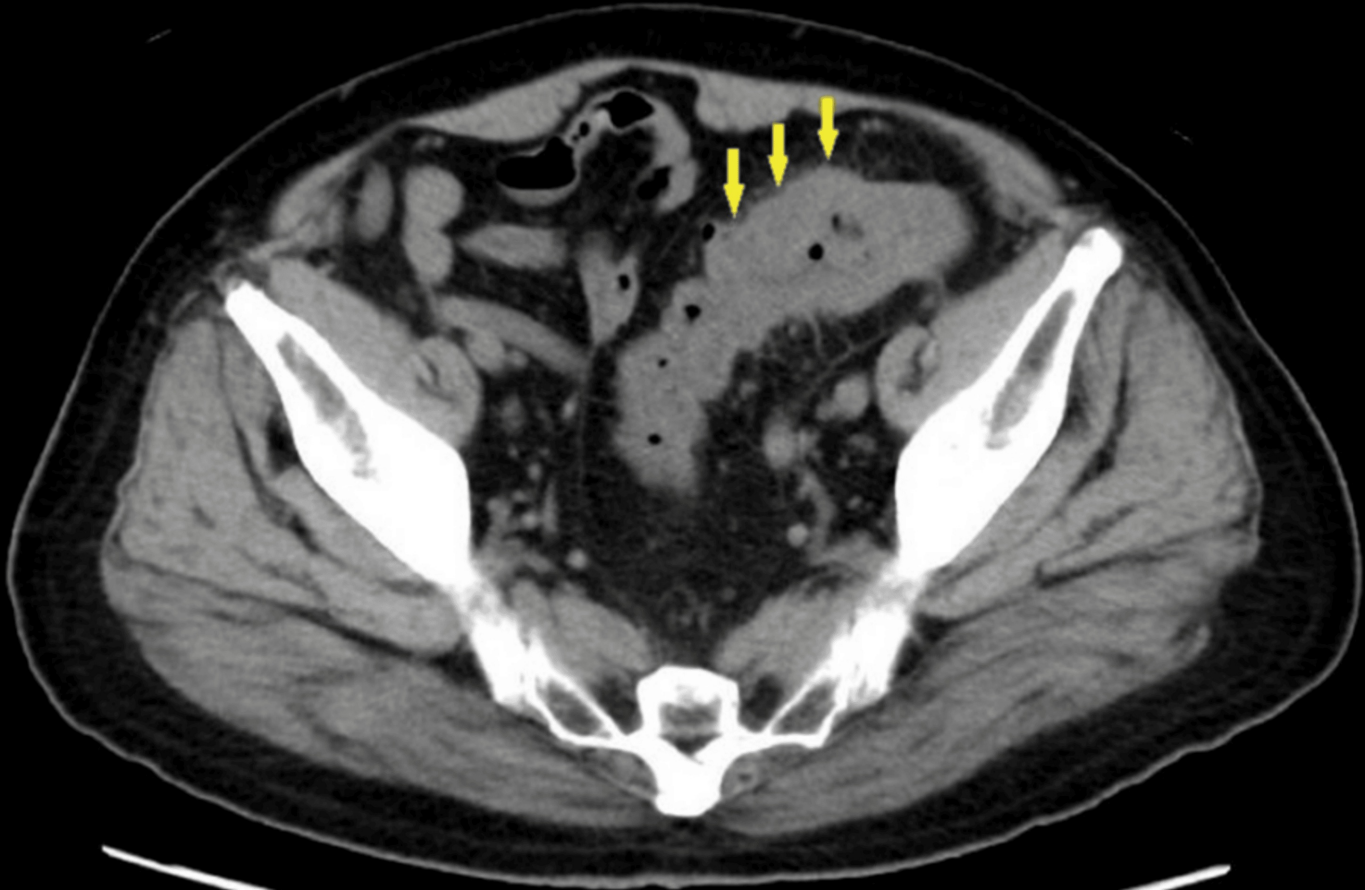
Plain abdominal CT scan Wall thickening of the sigmoid colon was apparent (yellow arrow).

The patient was an 87-year-old man with a history of atrial fibrillation, cerebral infarction, and positive anti-HBc antibody positivity. In year X-14, he began experiencing mucus and bloody stools. The following year (X-13), he was diagnosed with UC due to lesions in the sigmoid colon. Five-aminosalicylate medications (2,250 mg/day) and multiple courses of antibiotics proved ineffective. Prednisolone was administered in year X-12 (40 mg/day) but also failed to control his symptoms. However, tacrolimus was later given for several months (two or three months) and successfully resulted in remission. Tacrolimus was eventually discontinued in favor of herbal therapy, which appeared to be beneficial in reducing symptoms. Colonoscopy in year X-2 revealed mucosal edema and bleeding in the sigmoid colon, 25 cm from the anal verge (Figure 1a). The lesion was attributed to a diverticulum. Diarrhea and anemia continued in year X-1. A colonoscopy showed mucosal edema, stenosis, and deep ulcers in the sigmoid colon (Figure 1b). Biopsies from the ulcers revealed no malignancy. Abdominal CT scan indicated wall thickening in the sigmoid colon, leading to antibiotic treatment for suspected diverticulitis. The most recent colonoscopy (year X) revealed an enlarged lesion in the sigmoid colon (Figure 1c). The rest of the colon appeared endoscopically free of active UC (Matts' endoscopic grade 1). A plain abdominal CT scan revealed wall thickening of the sigmoid colon (Figure 2). The preoperative diagnosis was diverticulitis with ischemic colitis. Due to the limited response to medical therapy (herbal medicine, transfusion, and levofloxacin), laparoscopic Hartmann's procedure was performed. Preoperative laboratory results showed hemoglobin 12.9 g/dL (13.5-17.6), albumin 3.8 g/dL (4.0-4.8), creatine kinase 382 U/L (30-200), and C-reactive protein 1.63 mg/dL (0-0.35). All other blood tests were within normal limits.

Pathological findings of surgical specimen

**Figure 3:**
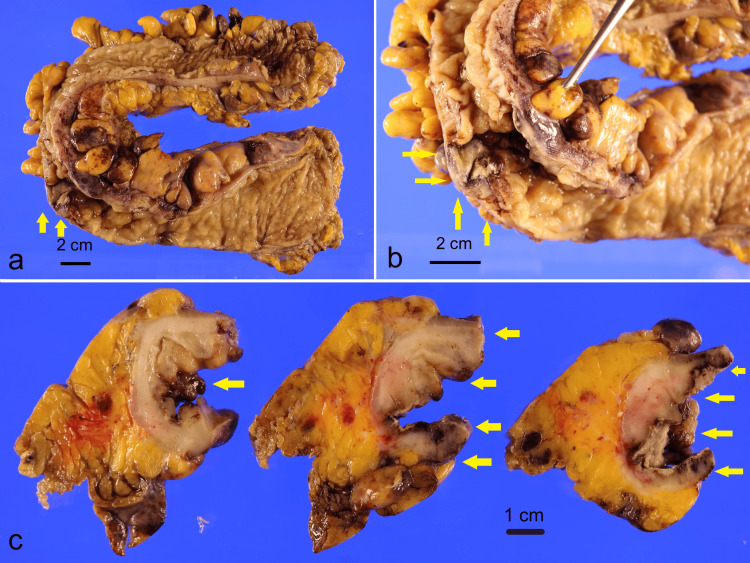
Specimens from Hartmann's procedure Macroscopically, the mucosa of the sigmoid colon protruded like a polyp and formed an ulcer covered with white moss (a, b, yellow arrow). In the cut surface, the layered structure of the intestinal wall got unclear, forming a white thickened lesion (c, yellow arrow).

The transverse, descending, and sigmoid colon and rectum were resected, extending proximally to 4 cm from the anal verge. Figures 3a-3c show the macroscopic appearances. The serosal surface appeared unremarkable with no perforations identified. Lymph nodes were visible within the subserosal layer.

**Figure 4:**
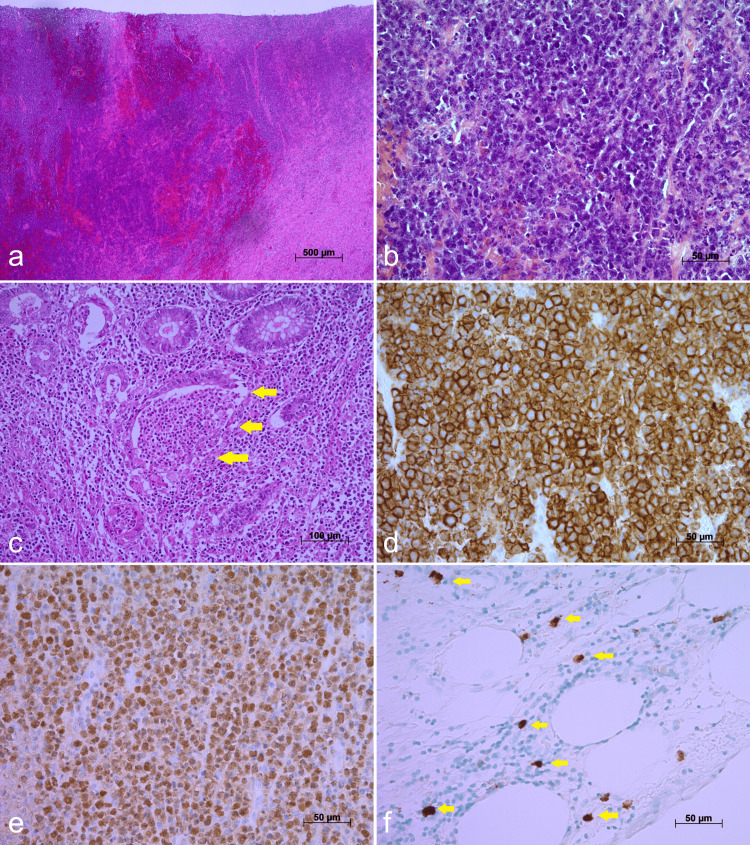
Microscopic images of the sigmoid colon (a) HE staining, original magnification 20 ×, scale bar = 500 µm. (b) HE staining, original magnification 200 ×, scale bar = 50 µm. (c) HE staining focusing on crypt abscesses (yellow arrow). Original magnification 100 ×, scale bar = 100 µm. (d) IHC for CD20, original magnification 200 ×, scale bar = 50 µm. (e) Epstein-Barr encoding region in situ hybridization, original magnification 200×, scale bar = 50 µm. (f) IHC for cytomegalovirus (yellow arrow), original magnification 200×, scale bar = 50 µm. HE, hematoxylin and eosin; IHC, immunohistochemistry

Histological examination revealed extensive ulceration with sloughed mucosal epithelium. The luminal surface was covered by inflammatory exudate and granulation tissue containing large numbers of neutrophils. Medium-to-large lymphoid cells displayed diffuse proliferation throughout the deep mucosa, extending to the superficial subserosal layer (Figures 4a, 4b). These cells exhibited monomorphic features, mitotic figures, and necrosis. Notably, Hodgkin and Reed-Sternberg cells were absent. Ulceration was accompanied by crypt distortion, goblet cell depletion, crypt abscesses (Figure 4c), and basal plasmacytosis. Lymphoid cell infiltration was predominantly confined to the mucosa and muscularis propria, with no involvement of subserosal lymph nodes identified. A pseudodiverticulum was observed near the ulcer. Distance mucosal areas displayed minimal inflammatory cell infiltration and lacked any specific findings.

Immunohistochemical analysis revealed positivity of the lymphoid cells for CD20 (Figure 4d), CD10, Bcl-6, and PD-L1 (22C3), while they were negative for CK AE1/AE3, CD3, Bcl-2, CyclinD1, MUM1, CD30, and CD15. The Ki-67 proliferation index was high, at 95%. In-situ hybridization confirmed the presence of Epstein-Barr encoding region within the lymphoid cells (Figure 4e). Scattered cytomegalovirus (CMV)-positive macrophages were also detected immunohistochemically (Figure 4f). Based on the histological and immunophenotypic features, the diagnosis was EBV-positive DLBCL. According to the Hans algorithm, this is classified as a germinal center B-cell subtype [12].

Postoperative course

PET/CT showed a right pelvic lesion after the surgery. Pola-R-CHP treatment was started (polatuzumab vedotin 110mg, rituximab 600mg, cyclophosphamide 800mg, doxorubicin 50mg, prednisolone 50mg). Seven months after the surgery, six courses of pola-R-CHP treatment were performed, and PET/CT showed a complete metabolic response.

## Discussion

We report the instructive case of an elderly UC patient with EBV-positive DLBCL following tacrolimus and herbal medicine therapy. Only one previous report described plasmablastic lymphoma in a UC patient treated with tacrolimus, but the case involved multiple medications, hindering a clear association with tacrolimus [13].

EBV-positive DLBCL arising in UC is rare. There have only been eight cases reported, including this one (Table 1) [1,14-19]. Notably, all eight patients were male, and many had lesions on the left side of the colon and rectum. Three cases reported bowel perforation. Ulcers and polypoid lesions are typical macroscopic and endoscopic findings. The case by Chang et al. involved a patient with polymyositis, and the treatment focused primarily on the polymyositis [16].

**Table 1:** Epstein-Barr virus-positive diffuse large B-cell lymphomas arising in ulcerative colitis 1) duration of ulcerative colitis (year) 2) site of malignant lymphoma 3) immunosuppressant and molecularly targeted therapy AZA, azathioprine; CMV, cytomegalovirus; CyA, cyclosporine; F, female; IFX, infliximab; M, male; MMF, mycophenolate mofetil; MTX, methotrexate; NA, not available; TAC, tacrolimus; 6-MP, 6-mercaptopurine

Author (year)	Sex	Age	Duration^1)^	Site^2)^	Gross change	Treatment^3)^	CMV
Schwartz et al. (2006) [14]	M	29	4	ileal pouch	polypoid mass	CyA, 6-MP, IFX	NA
Allen et al. (2013) [1]	M	65	5	sigmoid colon	perforation	AZA, 6-MP, IFX	NA
Hiyama et al. (2015) [15]	M	69	3.5	rectum	ulcer	AZA	NA
Chang et al. (2016) [16]	M	73	25	descending/sigmoid colon	polypoid/nodular area	MTX, MMF	NA
Suzuki et al. (2020) [17]	M	68	2.2	transverse colon to rectum	ulcer/multinodule/perforation	AZA, IFX	positive
Suzuki et al. (2021) [18]	M	43	8	rectum	ulcer/perforation	none	negative
Nakada et al. (2023) [19]	M	49	4	sigmoid colon	protruding lesion	none	NA
Our case	M	87	13	sigmoid colon	polypoid area/ulcer	TAC	positive

In our case, the patient with UC received tacrolimus for several months, and malignant lymphoma developed approximately 10 years after treatment cessation. The causal relationship was unclear. To our knowledge, no large-scale studies have clearly demonstrated the tumorigenicity of tacrolimus. There is also no consensus on the impact of treatment duration or time since treatment on lymphoma development in IBD patients receiving immunomodulators. A few UC cases reported lymphoma development even after short immunomodulator courses [14,17].

The present case involved an elderly immunocompromised patient with evidence of EBV and CMV infection. The diagnosis fell under lymphoproliferative disorder arising in immune deficiency and dysregulation, or EBV-positive DLBCL, not otherwise specified. It was reported that the rate of EBV infection increased with age in IBD patients [20], and aging may influence lymphoproliferation in our case. The 13-year interval between UC diagnosis and lymphoma suggests UC inflammation itself might have influenced lymphoma development due to local changes in the microenvironment. The herbal medicine mainly composed of indigo naturalis was taken for 12 years. Although there have been no reports of a relationship between malignant lymphoma and indigo naturalis, the influence of indigo naturalis cannot be completely ignored. Other differential diagnoses include ischemic enteritis, diverticulitis, UC exacerbation, and malignant tumors. This case highlights the challenge of diagnosing lymphoma before surgery.

## Conclusions

This case demonstrates malignant lymphoma accompanied by UC, and the preoperative diagnosis was difficult. Malignant lymphoma should be included as a differential diagnosis when atypical ulcers or polypoid lesions are seen in an elderly patient with UC after a long disease period or receiving multiple medications. The cause of malignant lymphoma was not completely clear, although several drugs and a long period of disease were suggested. Further accumulation of cases is necessary.
